# Antibacterial Properties of Crotoxin from *Crotalus durissus terrificus*—Insight into the Mechanism of Action

**DOI:** 10.3390/molecules27227726

**Published:** 2022-11-10

**Authors:** Dorota Nemecz, Patrycja Golińska

**Affiliations:** 1 Department of Biochemistry, Faculty of Biological and Veterinary Sciences, Nicolaus Copernicus University, 87-100 Torun, Poland; 2 Department of Microbiology, Faculty of Biological and Veterinary Sciences, Nicolaus Copernicus University, 87-100 Torun, Poland

**Keywords:** crotoxin, CB subunit, antibacterial activity, *Micrococcus luteus*, NlpC/P60 family

## Abstract

The growing problem of antibiotic resistance among bacteria requires searching for new therapeutic agents with bacteriostatic and/or bactericidal properties. Crotoxin is a β-neurotoxin from the venom of the *Crotalus durissus terrificus.* It is composed of two subunits: CA (non-active) and CB (with phospholipase A_2_ activity). It has already been shown that the isolated CB, but not the CA, subunit of crotoxin exhibits an antibacterial activity towards a variety of Gram-positive and Gram-negative bacterial species. However, no studies on the whole crotoxin complex have been carried out so far. We tested the antibacterial properties of crotoxin, as well as its isolated CB subunit, towards *Staphylococcus aureus* ATCC 25923, *Staphylococcus aureus* ATCC 6535, *Micrococcus luteus* ATCC 10240, *Escherichia coli* ATCC 25922, *Escherichia coli* ATCC 8739, and *Pseudomonas aeruginosa* ATCC 10145. Both toxins exhibited antibacterial properties only against *Micrococcus luteus* ATCC 10240. Crotoxin showed only bacteriostatic activity with a MIC of 46 µM, while the CB subunit acted as both a bacteriostatic and bactericidal agent with a MIC = MBC = 0.21 μM. The bacteriostatic effect of the toxins was independent of the enzymatic activity of the CB subunit. Bactericidal properties, however, require phospholipase A_2_ activity. Both toxins reduced bacteria viability at the MIC by 72% and 85% for crotoxin- and CB-treated bacteria, respectively. The membrane permeability increased approximately three times within the first hour of incubation with toxins; afterwards, either no significant changes or a decrease of membrane permeability, compared to the control cells, were observed. We isolated a single, approximately 30 kDa bacterial wall protein which belongs to the NlpC/P60 family that interacts with crotoxin leading to the inhibition of bacterial growth. Neither crotoxin nor the CB subunit showed any cytotoxic properties to human fibroblasts at the MIC during the three-day incubation.

## 1. Introduction

Snake venoms are complex mixtures of biologically active molecules, of protein and non-peptide origin, which exhibit a wide range of pharmacological properties [1,2]. It has been reported that both whole venoms as well as isolated venom components reveal an antibacterial activity which may be a new source of antibacterial agents. One of the major proteins responsible for the bactericidal properties of snake venoms are phospholipases A_2_ (PLA_2_) which are one of the three most common enzyme families in *Viperidae* venoms [3]. It has been shown that they may increase the permeabilization of the membrane and lead to bacterial cell death, both in an enzymatic and non-enzymatic way [4,5].

The *Crotalus durissus terrificus* (*Cdt*) is a South American rattlesnake belonging to the *Viperidae* family. The major toxin present in the venom of this snake is crotoxin [6,7]. This potent β-neurotoxin inhibits acetylcholine release by interacting with specific receptors at the presynaptic level of the neuromuscular junction and leads to muscle paralysis and death by asphyxia after snake envenomation [8,9]. Crotoxin is a heterodimeric protein composed of a basic PLA_2_ (CB subunit) and an acidic PLA_2_-like protein with no catalytic activity (CA subunit). Both subunits are essential for the neurotoxic activity of crotoxin, where the CA subunit acts as a chaperone and potentiates the toxic effect of the CB subunit [6]. Apart from neurotoxicity, crotoxin, as well as its PLA_2_ subunit, has also been shown to have other biological effects, such as cytotoxic [10,11,12], anti-inflammatory [13,14], analgesic [15,16], antiviral [17,18], and antibacterial effects [19,20,21]. The antibacterial effects aspect is very important because of the increasing antibiotic resistance among bacteria observed all over the world. This serious problem requires a new strategy in the field of health protection. Every year new multi-drug resistant strains (including resistant to last-line antibiotics) appear, causing a number of hard-to-cure infections, often leading to death, as currently available methods are not sufficient to combat them. Therefore, there is a great need to search for new therapeutic agents with bacteriostatic and/or bactericidal properties [22].

Various publications have shown that both whole venom [23] as well as an isolated CB, but not CA, subunit of crotoxin exhibits antibacterial activity towards a variety of Gram-positive and Gram-negative bacterial species [19,20,21]. Antibacterial activity seems to be independent from the PLA_2_ activity of the CB subunit, since its inhibition did not abolish bactericidal properties against *E. coli* [21]. However, no studies on the whole crotoxin complex have been carried out so far, and very little, if any, characterizations of the CB subunit’s antibacterial properties have been performed.

Therefore, we decided to test the antibacterial properties of the whole crotoxin complex (CACB) in addition to its isolated PLA_2_ subunit (CB) towards Gram-positive (*Staphylococcus aureus* ATCC 25923, *Staphylococcus aureus* ATCC 6535, and *Micrococcus luteus* ATCC 10240) and Gram-negative (*Escherichia coli* ATCC 25922, *Escherichia coli* ATCC 8739, and *Pseudomonas aeruginosa* ATCC 10145) bacteria in an effort to determine if the presence of the CA subunit in the crotoxin complex could affect these properties. Based on our results we were able to perform a basic characterization of the non-catalytic mechanism of action of crotoxin and the CB subunit on the bacterial cells. We believe that our results can be useful to design new drugs that would have a high therapeutic value.

## 2. Results

### 2.1. Crotoxin Shows Bacteriostatic and the CB Subunit Bactericidal Properties (Determination of MIC and MBC Values)

From all six tested bacterial strains, crotoxin and the CB subunit exhibited the strongest antibacterial properties against *Micrococcus luteus* ATCC 10240 in the used toxins’ concentration range. Crotoxin inhibited the growth of *M. luteus* at a minimal inhibition concentration (MIC) of 46 μM; however, bactericidal properties were not observed and, therefore, a minimal bactericidal concentration (MBC) was not determined. The subunit CB showed both growth inhibition and bactericidal properties with a MIC and MBC of 0.21 μM. Additionally, the presence of a PLA_2_ activity inhibitor did not fully abolish antibacterial properties of the CB subunit. The protein still inhibited bacterial growth at a MIC of 0.21 μM; however, the loss of bactericidal properties was observed. In addition, a weak growth inhibition of *Staphylococcus aureus* ATCC 25923 was observed after treatment with 10 μM (and higher) of the CB subunit. However, the MIC value could not be determined in this case.

### 2.2. Crotoxin Reduces Viability and Affects the Bacteria Membrane Permeability

**Figure 1:**
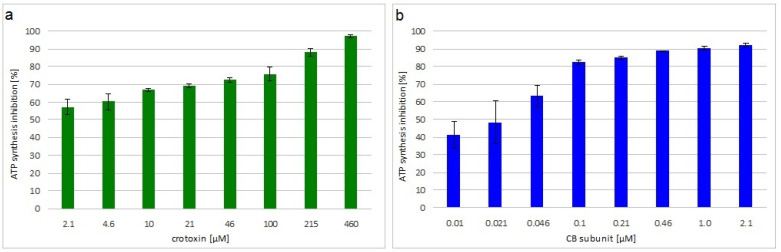
Viability of toxin incubated *M. luteus*. Average percentage inhibition of ATP synthesis by incubation with different concentrations of: (**a**) crotoxin or (**b**) CB subunit, relative to control, non-treated, cells. Error bars represent the standard deviation of three replicates.

The viability of *M. luteus* treated with crotoxin or the CB subunit was analyzed by measuring the level of ATP synthesis in the bacterial cells. Both toxins greatly decreased bacteria viability in a concentration dependent manner. Inhibition of ATP synthesis at the MIC was 72% and 85% for crotoxin- and CB-treated bacteria, respectively (Figure 1).

**Figure 2:**
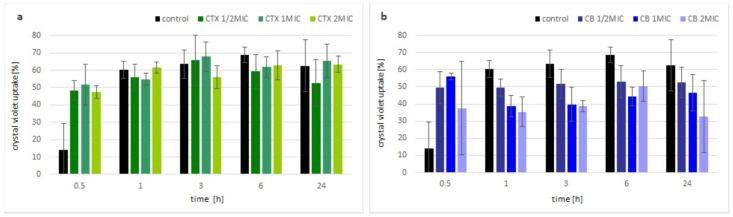
Membrane permeability of toxin incubated *M. luteus.* Time-dependent percentage of crystal violet uptake of treated cells with various concentrations of: (**a**) crotoxin, (**b**) CB subunit, in different time points. Error bars represent the standard deviation of three replicates.

The change in membrane permeability was analyzed by the uptake of the crystal violet dye. Within the first hour of incubation with crotoxin, the uptake of the dye by bacterial cells was visibly higher compared with the control (non-treated) bacteria, but independent of the applied concentration of toxin. After that time, there were no significant differences observed between the bacteria treated with crotoxin and the control culture, regardless of the used concentrations of crotoxin (Figure 2a). In the presence of the CB subunit, an increase in crystal violet uptake was observed up to the 1 h, similar to the crotoxin. However, further incubation of bacteria with the CB subunit resulted in almost no change (½MIC) or even slight decrease in the uptake of the crystal violet (MIC and 2MIC) (Figure 2b).

### 2.3. Crotoxin, but Not the CB Subunit, Interacts with a Bacteria Cell Wall Protein

**Figure 3:**
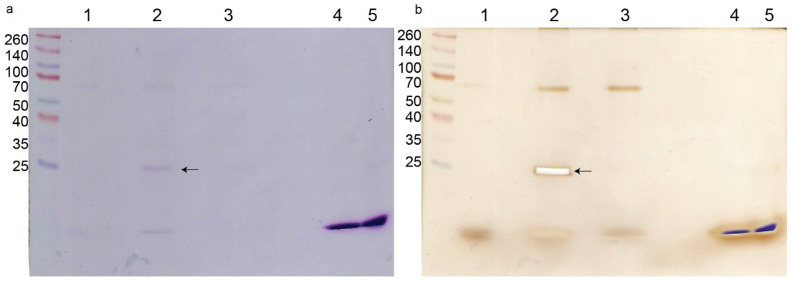
*M. luteus* protein interactions with crotoxin. SDS-PAGE of proteins separated in the pull-down experiment. (**a**) Coomassie staining, (**b**) silver staining. Lanes are: MW—molecular weight marker; 1—control cells (non-treated); 2—bacterial cells treated with crotoxin; 3—bacterial cells treated with CB subunit; 4—crotoxin alone; 5—CB subunit alone. Arrow indicates the protein interacting with crotoxin that was sent for analysis.

The pull-down experiment with biotinylated crotoxin resulted in the separation of a single protein (in addition to crotoxin itself) visible on an SDS-PAGE gel, compared with the non-treated bacterial cells (Figure 3a). This protein was identified by trypsin digestion and LC-MS/MS as a peptidoglycan endopeptidase belonging to the NlpC/P60 family with the molecular mass of 29,904 Da. There was no visible difference between the biotinylated CB subunit sample and non-treated bacteria even after silver staining (Figure 3b). Additionally, in all bacterial samples a non-specific ~68 kDa protein was visible on the gel.

### 2.4. Crotoxin, as Well as the CB Subunit, Do Not Show a Cytotoxic Activity towards Human Cells at the MIC

**Figure 4:**
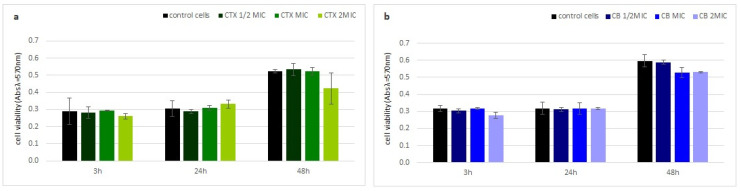
Toxin cytotoxicity on human cells. Time dependent fraction of viable human fibroblast cells (HDF) after incubation with (**a**) crotoxin and (**b**) the CB subunit relative to non-treated cells using the MTT test. Error bars represent the standard deviation of three replicates.

Neither crotoxin nor the CB subunit showed any cytotoxic properties to human fibroblasts (HDF). The viability of the HDF cells, measured by a MTT test, was similar to the control cells at every analyzed time point, even at the highest toxin concentration (Figure 4).

## 3. Discussion

Snake venoms are widely studied by researchers due to their richness of biological molecules, which exhibit numerous therapeutic properties, including antibacterial activity [24,25]. Searching for new antibacterial agents with new mechanisms of action is important due to a growing problem of microorganisms’ drug resistance around the world [22,26].

### 3.1. Crotoxin Is Not a Main Antibacterial Agent in the Venom of Crotalus durissus terrificus

In our study crotoxin and the CB subunit showed the strongest antibacterial properties towards *Micrococcus luteus*. Additionally, the CB subunit exhibited a weak bacteriostatic activity against *S. aureus* ATCC 25923. The antibacterial activity of crotoxin and the CB subunit against other tested bacterial strains, namely *S. aureus* ATCC 6538, *E. coli* ATCC 25922, *E. coli* ATCC 87392, and *P. aeruginosa* ATCC 10145, was not observed at the tested concentration range. It has been reported previously that the crude venom of *Cdt* has a strong antibacterial activity against *S. aureus* ATCC 25923, *P. aeruginosa* ATCC 27853, and *M. luteus* ATCC 9341 [23]. Since, among snake venom components, PLA_2_ was reported as one of the main antibacterial agents, crotoxin could be a good candidate to be responsible for that effect [5]. However, our results are not fully consistent with this hypothesis. Crotoxin showed no activity and the CB subunit only a very low bacteriostatic effect against *S. aureus*. Additionally, we did not observe any activity against *P. aeruginosa*. These results would suggest that neither crotoxin nor the CB subunit alone were responsible for the antibacterial action on this bacterium, and that another *Cdt* venom component must play a key role in this process.

Our study corroborates strong activity against *M. luteus* by crotoxin and the CB subunit; however, the bacteria strain (ATCC 9341) used for the experiments with the crude venom was reclassified by Tang and Gillevet, in 2003, to the *Kocuria rhizophila* strain based on combined molecular, cytochemical, and physiological characteristics [27]; therefore, those results cannot ultimately be compared with our data.

### 3.2. Antibacterial Properties of Crotoxin and the CB Subunit Depend on the Bacteria Strain, Not the Species

The isolated CB subunit was indicated in other reports as an antibacterial agent against different bacterial species, although, in those papers, different bacterial strains [21] or clinical isolates [19,20] were tested. It was previously presented that the isolated CB subunit revealed antibacterial properties against *E. coli* ATCC 29648 [21]. Our results are not inconsistent with these data. We did not observe any effect of the CB subunit as well as crotoxin against two tested strains of *E. coli* (ATCC 8739 and ATCC 25922). Although, in our study, we used lower concentrations (0.01–46 µM) of the CB subunit compared with the aforementioned study (40–100 µM), a comparison between the lower range 40 µM and our maximal 46 µM may be made. Therefore, we suggest that the differences between these observations may result from the differences between the tested bacteria strains, rather than the species. This theory is supported by another experiment, in which the CB subunit showed strong antibacterial properties towards a clinical isolate of *E. coli* at a concentration of 7 μM [20]. A similar situation is observed for the results with *P. aeruginosa*. Our experiments, carried out using the strain ATCC 10145, did not show any antibacterial properties of either crotoxin or the CB subunit in the concentration range of 2.1–460 μM and 0.01–46 µM, respectively. However, the clinical isolate of this bacteria species was sensitive to the isolated CB subunit at a concentration of 7 μM [20]. Based on these observations, we suggest that sensitivity to this antimicrobial compound depends on specific properties of individual bacterial strains and not simply the species.

### 3.3. Crotoxin Does Show Antibacterial Properties in Both an Enzymatic and Non-Enzymatic Manner

Crotoxin inhibited only the growth of *M. luteus* while the CB subunit acted as both a bacteriostatic and a bactericidal agent. It seems that the presence of the CA subunit in the crotoxin complex reduces the toxicity against bacteria. During snake envenomation, the CA subunit enables the CB subunit to interact with a specific receptor and increases its toxicity by preventing interactions with other molecules [28]. Here, we show that the CA subunit plays an opposite role in the antibacterial action. These results are consistent with the determined MIC values as the whole crotoxin complex (CACB) exhibited much weaker (220-fold) bacteriostatic properties against *M. luteus* compared with the CB subunit alone (MIC 46 μM and 0.21 μM, respectively). The results obtained in the viability tests also indicate that the CA subunit inhibits antibacterial properties of the CB subunit. On the other hand, the CA subunit naturally blocks enzymatic activity of the CB subunit in the crotoxin complex [28], suggesting that the antibacterial properties of the CB subunit would greatly depend on its PLA_2_ activity. However, bacteriostatic properties of the CB subunit were not abolished by the presence of a PLA_2_ inhibitor. Therefore, the inhibition of bacterial growth occurs independently of the enzymatic activity. These results are consistent with the paper of Soares et al. (2001) [21] which shows that various modifications, which lead to the inhibition of catalytic activity (such as alkylation of a His residue by BPB or incubation with EDTA), of the CB subunit did not affect bactericidal activity against *E. coli*. However, according to our data, the bactericidal effect of this protein required the phospholipase activity, since the PLA_2_ inhibitor fully blocked this property. It is believed that antibacterial properties of snake venom PLA_2_, which are independent from the catalytic activity, are due to the specific combination of hydrophobic and cationic residues at the C-terminal of the polypeptide chain, which interact with the bacterial membrane causing its disturbance [5]. Taking all of our results together, it seems that the antibacterial action of crotoxin involves mostly a region located in the CB subunit which is partly covered by the CA subunit in the complex. Furthermore, according to the earlier studies of other snake venom PLA_2_s, a peptidic fragment of the C-terminal region of the protein, consisting of 10–22 amino acids, is responsible for some antibacterial effects. This suggests that the C-terminal region of the CB subunit, of which the residues 118–121 are occluded by the CA subunit [29], could be associated with an antibacterial activity.

Numerous publications have shown that snake venom PLA_2_ can act via their phospholipase activity or in a non-enzymatic manner increasing the permeability of the cell membrane and causing the bacterial cell wall disintegration [5].

In order to characterize the mechanism of action by crotoxin and the CB subunit on bacteria, we performed a crystal violet test and a pull-down experiment. Crystal violet was previously reported as a dye that hardly penetrates the cell membrane of healthy cells but can easily accumulate in the cells with disrupted membranes [30]. Our experiment showed that within the first hour of bacterial incubation with crotoxin or the CB subunit, the uptake of the dye is higher compared with the control, non-treated bacteria cells. These results suggest some destabilization of the bacterial membrane in the response to crotoxin or the CB subunit. However, over time, the amount of dye uptake did not increase or even decreased in the presence of the CB subunit, whereas the dye uptake by control cells was increasing, suggesting the reduction of the bacterial cell membrane permeability. Antibacterial action of crotoxin, which results from bacterial cell wall alteration, was partially confirmed by the pull-down experiment from which we isolated a single ~30 kDa protein that belongs to the NlpC/P60 family. This large and diverse family of proteins is present in all bacteria and contains peptidoglycan-hydrolyzing enzymes that are involved in bacterial cell wall dynamics [31]. Bacterial cell wall hydrolases (BCWH), including lysozymes, autolysins, and virolysins, have been already suggested as potential antibacterial agents that can be novel alternatives to antibiotics [32]. Peptidoglycans that build the bacterial cell wall are responsible for its rigidity. Interaction of crotoxin with the *M. luteus* peptidoglycan peptidase can disrupt the reorganization of the bacterial wall during their growth and division, causing the bacteriostatic effect.

## 4. Conclusions

In conclusion, crotoxin does not seem to be the major antimicrobial agent of the *Cdt* venom. Its, as well as the CB subunit’s, antibacterial properties may differ between bacterial strains of the same species. The bacteriostatic effect of the toxins was independent of the enzymatic activity of the CB subunit; however, bactericidal properties require PLA_2_ activity. It seems that non-enzymatic inhibition by crotoxin/the CB subunit occurs through the interaction with an N1pC/P60 protein which is responsible for cell wall reorganization during bacterial growth. Furthermore, neither the CB catalytic subunit nor crotoxin showed cytotoxic effects against human fibroblast cells. The above results indicate that the peptidic binding region of crotoxin with the N1pC/P60 family of proteins could potentially be used in further studies as a therapeutic agent preventing bacterial infections.

## 5. Materials and Methods

### 5.1. Crotoxin, CB Subunit

Crotoxin as well as its isolated subunit (as previously described [33]) were a kind gift from Dr. Grazyna Faure from the Pasteur Institute in France. The proteins were stored at 4 °C in the form of lyophilized powder and dissolved prior to an experiment in TSB medium, H_2_O, or PBS buffer (depending on the protocol) at the desired concentration.

### 5.2. Bacteria Strains

In this study, both Gram-positive (*Staphylococcus aureus* ATCC 6538, *Staphylococcus aureus* ATCC 25923, *Micrococcus luteus* ATCC 10240), and Gram-negative (*Escherichia coli* ATCC 8739, *Escherichia coli* ATCC 25922, *Pseudomonas aeruginosa* ATCC 10145) bacteria were used. For experimental use, bacteria were grown in Triptic Soy Broth (TSB, Becton Dickinson, Franklin Lakes, NJ, USA) for 24 h at 37 °C under shaking conditions (120 rpm).

### 5.3. Determination of Minimal Inhibitory Concentration (MIC) and Minimal Bactericidal Concentration (MBC)

MICs of crotoxin or the CB subunit were determined in triplicate using the serial two-fold micro-dilution technique in 96-well plates according to the Clinical and Laboratory Standards Institute (CLSI). The used crotoxin and the CB subunit concentration ranges were 2.1–460 μM and 0.01–46 μM, respectively. Crotoxin or the CB subunit solutions in TSB were inoculated with bacterial strains in a final density of 10^5^ CFU/mL. Bacteria cultured without crotoxin or the CB subunit served as a positive control, while the TSB medium itself was treated as a negative control. The plates were incubated for 24 h at 37 °C. The MIC value was defined as the lowest concentration of toxin which visibly inhibited bacterial growth.

Afterwards, to determine the MBC value, 100 μL of treated bacterial cultures (at MIC and above) were spread on Triptic Soy Agar (TSA, Becton Dickinson, Franklin Lakes, NJ, USA) plates and incubated for 24 h at 37 °C. The MBC value was taken to be the concentration of the toxin at which ≥99.99% bacterial growth was inhibited after 24 h incubation of treated bacteria on agar plates.

### 5.4. Effect of PLA_2_ Activity Inhibitor on Antibacterial Properties

The effect of the LY311727 inhibitor on antibacterial properties was tested for the CB subunit only, analogously to the MIC experiment, with the CB subunit concentration range of 0.01–2.1 μM and 200 μM inhibitor in DMSO. Bacteria treated with inhibitor or DMSO only served as additional controls.

### 5.5. Determination of Bacterial Viability

Viability of bacteria treated with crotoxin or the CB subunit was analyzed by the quantitative determination of ATP using a BacTiter-Glo™ kit (Promega, Walldorf, Germany), according to the manufacturer’s protocol. The experiment was performed in 96-well plates in triplicate. Bacteria at the density of 10^7^ CFU/mL were incubated with crotoxin (2.1–460 μM) or the CB subunit (0.01–2.1 μM) for 5.5 h at 37 °C in a total volume of 100 μL. Subsequently, 50 μL of the incubation mixture from each well was mixed with 50 μL of BacTiter-Glo reagent in an opaque 96-well plate and incubated for 5 min at room temperature. The luminescence of the samples was measured at 600 nm using a Multidetection Spectramax iD3 reader (Molecular Devices, San Jose, CA, USA).

### 5.6. Crystal Violet Assay

For the crystal violet experiment, bacteria at a density of 10^8^ CFU/mL were centrifuged for 5 min at 4000× *g*, washed twice, and resuspended in PBS. The cells were incubated with crotoxin or the CB subunit at ½MIC, MIC, or 2MIC at 37 °C under shaking conditions. The control sample, without toxins, was prepared similarly. After incubation for 0.5, 1, 3, 6, and 24 h, 0.5 mL of the culture was transferred to a new clean tube and centrifuged for 5 min at 4000× *g*. The cell pellet was incubated in PBS containing crystal violet (10 μg/mL) for 10 min at 37 °C and then centrifuged again for 5 min at 4000× *g*. A spectrophotometric measurement of the supernatant was performed at λ = 590 nm using an Epoch microplate spectrophotometer (BioTek Instruments, Winooski, VT, USA). The absorbance value of the crystal violet solution was treated as 100%. The percentage of crystal violet uptake was calculated using the formula below:

[1 − (OD590 of the sample/OD590 of the crystal violet solution)] × 100

Each variant was tested in triplicate.

### 5.7. Pull-Down Experiment

Crotoxin and the CB subunit were biotinylated using EZ-Link NHS-Biotin Reagent (Thermo Scientific, Waltham, MA, USA) according to the manufacturer’s protocol. Each protein was mixed with 12-fold molar excess of biotin and incubated for 30 min at room temperature. To remove the non-reacted biotin, both protein solutions were dialyzed overnight at 4 °C against PBS. The following day, the biotinylated crotoxin or CB subunit was added to a 10 mL bacterial culture at the concentration of ½MIC and incubated for 3 h at 37 °C under shaking conditions. Non-treated bacteria served as the control. Afterwards, bacteria were centrifuged for 10 min at 5000× *g* at room temperature. The pellet was washed twice with PBS and resuspended in 1 mL of PBS containing 15 mM sodium cholate and 1 mM PMSF, followed by an incubation with 100 μL of Dynabeads MyOne Streptavidin T1 (Invitrogen, Waltham, MA, USA) for 30 min at room temperature. Subsequently, the beads were washed three times with PBS and then resuspended in SDS-PAGE sample buffer.

### 5.8. SDS-PAGE and Gel Staining

The electrophoresis was performed using the Ogita and Markert method [34]. Protein separation was performed using a C.B.S. Scientific mini vertical electrophoresis system, and 4% stacking/12% running gels (8 × 8 cm). Samples were loaded on the gel after incubation for 10 min at 100 °C and centrifugation took place on a mini centrifuge at 5000× *g* for 3 min. After electrophoresis, the gel was stained overnight with 0.1% Coomassie Brilliant Blue R-250 solution followed by discoloration with a solution composed of 35% methanol and 10% acetic acid. For the silver staining, the Coomassie stained gel was incubated overnight in a solution containing 50% ethanol and 12% acetic acid, followed by 3 × 20 min in 50% ethanol, and 1 min in 0.002% sodium thiosulfate (Na_2_S_2_O_3_). After intensive washing with water (3 × 20 s), the gel was incubated 2 × 20 s in 0.2% silver nitrate (AgNO_3_) solution containing 0.75 mM formaldehyde (HCHO), followed by a solution containing 6% sodium carbonate (Na_2_CO_3_), 0.02% sodium thiosulfate, and 0.00005% formaldehyde until visible brown stripes appeared. The reaction was stopped with a solution containing 50% ethanol and 12% acetic acid.

### 5.9. Protein Identification by LC-MS/MS

Identification of the protein separated in a pull-down experiment was carried out in the Environmental Mass Spectrometry Laboratory, Institute of Biochemistry and Biophysics Polish Academy of Sciences in Warsaw, Poland. The protein band was cut out from the gel and proteolytically degraded using trypsin. Obtained peptides were separated by liquid chromatography (LC) and the mass measurement of peptides and their fragments was performed in a mass spectrometer using the Orbitrap spectrometer (Thermo).

### 5.10. MTT Test

Human skin fibroblasts (HDF cell line) were cultured in a low-glucose DMEM medium with 10% FBS at 37 °C and 5% CO_2_. Cells were seeded on a 48-well plate at a density of 10^4^ cells/well and cultured for 24 h under standard culture conditions. Subsequently, the medium was replaced with a medium containing crotoxin or the CB subunit at ½ MIC, MIC, and 2MIC. After 3, 24, and 48 h of incubation the medium was removed and 150 μL of MTT reagent solution in the medium (0.5 mg/mL) was added to the wells, followed by a 45 min incubation at 37 °C, and washing with PBS. Obtained formazan crystals were dissolved in 150 μL of DMSO. Spectrophotometric measurement was performed at λ = 570 nm using an Epoch microplate spectrophotometer (BioTek Instruments, Winooski, VT, USA). The results are shown as the fraction of cell vitality in relation to the control, non-treated culture. The experiment was performed in triplicate.

## Data Availability

All data generated or analyzed during this study are included in this published article, any questions may be addressed to the corresponding author.
